# 2843. Risk Factors for Unplanned Healthcare Encounters in Patients Discharged from the Emergency Department with *Extended-spectrum β-lactamase* Urinary Tract Infections

**DOI:** 10.1093/ofid/ofad500.2453

**Published:** 2023-11-27

**Authors:** Jessica M Brochu, Rachel M Kenney, Shelbye Herbin, Satheesh Gunaga, Michael Veve

**Affiliations:** Henry Ford Hospital, Detroit, Michigan; Henry Ford Hospital, Detroit, Michigan; Henry Ford Wyandotte Hospital, Wyandotte, Michigan; Henry Ford Wyandotte Hospital / Envision Healthcare, Wyandotte, Michigan; Henry Ford Health, Detroit, Michigan

## Abstract

**Background:**

The management of extended spectrum β-lactamase (ESBL) urinary tract infections (UTIs) in the emergency department (ED) has not been well documented in literature. The purpose of this study was to describe treatment and outcomes of patients with ESBL UTIs in the ED, which can inform best practices for antimicrobial stewardship.

**Methods:**

This IRB approved, retrospective cohort analysis included patients who were discharged from the ED with an ESBL UTI between January 2020 and November 2022. The primary outcome of interest was any unplanned healthcare encounter related to the UTI within 30 days of the index ED visit, which included phone/virtual visits, clinic visits, ED visits, and hospitalizations. Patients ≥18 years of age treated for symptomatic UTI with a monomicrobial urine culture were included, while those with altered mental status, history of renal transplant, abnormal urinary tract, or were on active antibiotic therapy prior to the ED visit were excluded. Patient characteristics, initial and definitive antibiotic therapy, culture results including pathogen and susceptibilities were described. Logistic regression analysis was used to identify any exposures that were independently associated with unplanned healthcare encounters related to the UTI.

**Results:**

162 patients were included; 103 (64%) had an unplanned healthcare encounter. Patient characteristics are depicted in **Table 1**. Complicated lower UTI was most frequent occuring in 71 patients (44%). Susceptibility data revealed that nitrofurantoin was effective in 121 (75%) patients, aminoglycosides in 117 (72%) patients, TMP/SMX in 66 (41%) patients, and quinolones in 62 (38%) patients. 76 (74%) patients with unplanned encounters received an inactive empiric prescription: β-lactams being prescribed to 52 (51%) patients with cephalexin used for 49/52 (94%). Of the 81 patients with lower UTI, 20 (25%) initially received a prescription for nitrofurantoin. Factors associated with an unplanned healthcare encounter are described in **Table 2**.

**Figure:**
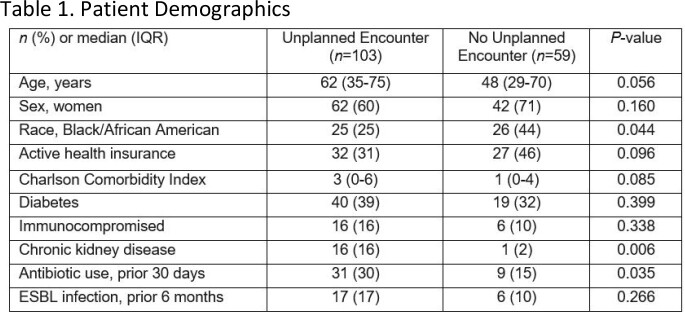

**Table 2.**
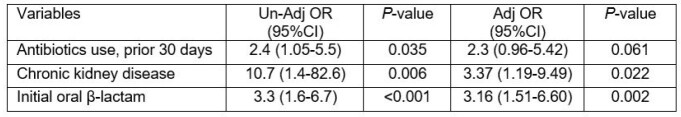
Factors Associated with Unplanned Healthcare Encounters. Abbreviations: Un-Adj OR = unadjusted odds ratio; Adj OD = adjusted odds ratio

**Conclusion:**

Patients discharged from the ED with an ESBL UTI were at higher risk for unplanned healthcare encounters if they had CKD or were initially prescribed an oral β-lactam. Prescribing first line therapy with nitrofurantoin for lower UTI is a potential area for improvement.

**Disclosures:**

**Michael Veve, PharmD, MPH**, National Institutes of Health: Grant/Research Support|Paratek Pharmaceuticals: Grant/Research Support

